# Do olfactory and gustatory psychophysical scores have prognostic value in COVID-19 patients? A prospective study of 106 patients

**DOI:** 10.1186/s40463-020-00449-y

**Published:** 2020-08-06

**Authors:** Luigi Angelo Vaira, Claire Hopkins, Marzia Petrocelli, Jerome R. Lechien, Damiano Soma, Federica Giovanditto, Davide Rizzo, Giovanni Salzano, Pasquale Piombino, Sven Saussez, Giacomo De Riu

**Affiliations:** 1 Maxillofacial Surgery Operative Unit, University Hospital of Sassari, Viale San Pietro 43/B, 07100 Sassari, Italy; 2 Guy’s Hospital, King’s College, London, UK; 3 Maxillofacial Surgery Operative Unit, Bellaria and Maggiore Hospital, AUSL Bologna, Bologna, Italy; 4 https://www.yoifos.com/; 5 Department of Human and Experimental Oncology, Faculty of Medicine UMONS Research Institute for Health Sciences and Technology, University of Mons (UMons), Mons, Belgium; 6 Otolaryngology Operative Unit, University Hospital of Sassari, Viale San Pietro 43/B, 07100 Sassari, Italy; 7 Maxillofacial Surgery Unit, University Hospital of Naples “Federico II”, Via Pansini 5, 80131 Naples, Italy

**Keywords:** COVID-19, Ageusia, Anosmia, Olfactory, Gustatory

## Abstract

**Background:**

The lack of objective data makes it difficult to establish the prognostic value of chemosensitive disorders in coronavirus disease 2019 (COVID-19) patients. We aimed to prospectively monitor patients diagnosed with COVID-19 to see if the severity of olfactory and gustatory dysfunction associates with subsequent disease severity.

**Methods:**

Multicentre prospective study that recruited 106 COVID-19 subjects at diagnosis. Chemosensitive functions were assessed with psychophysical tests within 4 days of clinical onset, at 10 and 20 days. Daily body temperature and oxygen saturation were recorded as markers of disease severity alongside need for hospitalisation. The correlation between olfactory and gustatory scores and disease severity was assessed with linear regression analysis.

**Results:**

At T0, 71 patients (67%) presented with olfactory dysfunction while gustatory impairment was detected in 76 cases (65.6%). Chemosensitive disorders gradually improved over the observation period. No significant correlations were found between T0 chemosensitive scores and final disease severity. The correlation between olfactory scores and fever proved significant at T2 (*p* = 0.05), while the relationship with gustatory scores was significant at T1 (*p* = 0.01) and T2 (*p* <  0.001), however neither was clinically relevant. The correlation between chemosensitive scores and oxygen saturation was significant only for taste at T2 (*p* <  0.001). Logistic regression analysis found significant correlations between olfactory impairment severity and need for hospitalization at T2 (OR 3.750, *p* = 0.005).

**Conclusions:**

Initial objective olfactory and gustatory scores do not seem to have a significant prognostic value in predicting the severity of the COVID-19 course; however, persistence of olfactory dysfunction at 20 days, associated with a more severe course. Unfortunately, olfactory and gustatory dysfunction do not seem to hold prognostic value at the time of initial diagnosis.

## Introduction

A growing evidence base has established a high prevalence of olfactory and gustatory disorders in patients affected by coronavirus disease 2019 (COVID-19) [1–9]. Most reports are anamnestic and few studies include psychophysical evaluation of the olfactory and gustatory functions [10–14]. The lack of such data makes it difficult to establish the prognostic value of chemosensitive disorders in COVID-19 patients. In fact, studies based only on patient interviews or on the analysis of medical records tend to underestimate the frequency of these symptoms [12], and there is a significant risk of recall bias in patients with severe disease, particularly those requiring ventilatory support and too unwell to eat or drink.

Based on data collected from the medical records of 169 COVID-19 patients, Yan et al. [15] reported that the presence of chemosensitive disorders is significantly associated with milder forms of COVID-19; in particular they stated that the development of anosmia was predictive of avoidance of the need hospital admission. In addition to the limitations related to its anamnestic nature, this study uses only the need for hospitalization as a clinical outcome. Although this was a single-centre study, this severity marker is not standardized as hospitalization criteria may vary with different patient groups according to age, co-morbidities and hospital capacity. These limitations may have important public health implications if such studies become the basis for establishing guidelines, and such reports may adversely influence patient behaviour in seeking medical care [16].

Vaira et al. [10, 12] and Moein et al. [13] objectively assessed COVID-19 patients with psychophysical tests without detecting a statistically significant correlation between chemosensitive scores and clinical severity. Although based on more objective data, these studies also have limitations in assessing the prognostic value of chemosensitive disorders; they assessed olfactory and gustatory function at different time points and retrospectively compared this with disease severity.

With the aim of overcoming these limitations, the present study prospectively assessed chemosensitive functions and clinical markers of 106 COVID-19 patients, for 20 days from symptom onset. We aimed to assess whether the severity of chemosensitive disorders could predict need for hospitalisation, and whether they associated with proxy markers of disease severity; oxygen saturation and fever. The statistical correlation between these parameters was therefore assessed in order to evaluate the prognostic value of the olfactory and gustatory scores.

## Materials and methods

This multicentre prospective study was carried out at the University Hospital of Sassari and the Maggiore-Bellaria Hospital in Bologna. The evaluation protocol was approved by an independent ethics committee (n° 378–2020-OSS-AUSLBO). All subjects were part of the hospitals’ healthcare staff and provided informed consent for participation in the study. To be enrolled in the study, patients had to comply with the following inclusion criteria: adults over 18 years of age, symptomatic patient with clinical onset for less than 4 days, confirmed diagnosis of SARS-CoV-2 infection in the nasopharyngeal swab.

The exclusion criteria included: psychiatric or neurological diseases, previous trauma, surgery or radiotherapy in the oral and nasal cavities, pre-existing taste or smell dysfunctions, history of allergic rhinitis or chronic rhinosinusitis, pre-existing hypoxic pulmonary disease, refusal to participate in the study.

### Anamnestic and clinical data

From enrolment, all patients were prospectively evaluated over a 20-day observation period. First, some anamnestic data were collected for all subjects: gender, age, comorbidities, possible causes of exclusion from the study. All patients were therefore asked to monitor daily two clinical parameters, associated with the severity of the disease [17, 18]: body temperature and oxygen saturation. As required by the Health Surveillance Department, these parameters were measured 3–4 times a day; for the purpose of the study, the mean of the measurements of each parameter was considered. If the subject the subject was hospitalized during the observation period, the clinical parameters were obtained from the medical records. If the patient was receiving oxygen therapy, saturation after 60 s in ambient air was measured and considered.

### Olfactory and gustatory data

The objective evaluation of the olfactory and gustatory functions with the psychophysical tests was performed at the beginning of the observation period (T0) and 10 (T1) and 20 days after the symptom onset (T2).

The self-administered olfactory and gustatory psychophysical test was used to evaluate patients in home quarantine. This patient self-administered telephone test is performed remotely by the operator and has recently been validated for the evaluation of patients in home quarantine using common house-hold odorants and flavors [11]. The test investigates the ethyl-alcohol olfactory threshold with nine solutions with increasing dilutions and the gustatory and olfactory discriminatory functions through seven groups of odorants and four flavoured solutions prepared directly by the patient. If the patient was hospitalized during the observation period chemosensitive functions were evaluated directly by the operator. Smell assessment was performed by means of the Connecticut Chemosensory Clinical Research Center (CCCRC) orthonasal olfaction test [19], a widely used ad validated test that includes that includes a butanol threshold evaluation and an odor identification task. A validated discrimination test was carried out to assess the taste function, investigating the discriminative capability for four primary tastes [20].

The evaluation methodology and the scoring system of the two tests have been previously described in detail [10–12].

### Statistical analysis

The two tests provided standardized data on the same evaluation scale of the olfactory and gustatory function that can be analysed together. Statistical analysis was performed using SPSS 26.0 (IBM, Armonk, NY, USA). Categorical variables are reported in numerals and percentages of the total. Descriptive statistics for quantitative variables are given as the mean ± standard deviation (SD). Linear regression analysis was used to evaluate the association between olfactory and taste scores (independent variables) and clinical severity markers (dependent variables). Logistic regression was instead performed to assess the association between the chemosensitive scores and comorbidities and the need for hospitalization during the observation period. Based on the scores obtained, the patients were divided into two groups: severe dysfunctions (anosmia or ageusia or severe and moderate hyposmia and hypogeusia) and normal (normal chemosensitive function or mild hypogeusia and hyposmia). Crosstab analysis and Fisher exact test were performed to calculate the odds ratio of the probability of hospitalization based on the severity of the chemosensitive dysfunction. The level of statistical significance was set at *p* ≤ 0.05 with a 95% confidence interval.

## Results

A total of 106 COVID-19 patients met the inclusion criteria and completed the evaluation. There were 53 males and 53 females. The mean age was 49.6 years old (interquartile range 43–55.2). General and clinical features of the patients are reported in Table 1. At T0, all subjects were in home quarantine. During the observation period 30 patients (28.3%) needed hospitalization due to the deterioration of respiratory function.

**Table 1:** General and clinical features of the study population

N° of patients (%)	
**Gender**	
Male	53 (50%)
Female	53 (50%)
**Age (years)** **mean (SD)**	49.6 (8.5) (IQR 43–55.2)
**Body temperature (°C)** **Mean (SD)**	37.7 °C (0.39)
**Oxygen saturation (%)** **Mean (SD)**	94.1% (1.9)
**Need for hospitalization** **N° of patients (%)**	30 (28.3%)
**Comorbidities**	
**> 2 comorbidities** **N° of patients (%)**	5 (4.7%)
**2 comorbidities** **N° of patients (%)**	7 (6.6%)
**1 comorbidity** **N° of patients (%)**	20 (18.9%)
**No comorbidities** **N° of patients (%)**	74 (69.8%)

### Olfactory function evaluation results

At T0, 71 patients (67%) presented with olfactory dysfunction. The reported alterations included mostly severe dysfunctions: anosmia in 44 patients and severe hyposmia in 11 cases. At the 10-day evaluation (T1), severe disorders continued to be the most frequent finding, with a severe anosmia and hyposmia frequency of 15.1 and 27.4%, respectively. No patient deteriorated between T0 and T1, or from T1 to T2. A more marked improvement was found at the 20-day control (T2), when the olfactory function was normal in 44.3% of patients. At the end of the observation period a severe olfactory disorder (i.e. anosmia or severe hyposmia) persisted in 19.7% of patients. Nine patients remained anosmic at T2 and all had been anosmic at the baseline assessment. Table 2 reports a summary of the olfactory evaluation results.

**Table 2:** Olfactory and gustatory assessment results. The table shows the average olfactory and gustatory scores and the frequencies of each type of dysfunction at the three observation times

**T0 olfactory score** **Mean (SD)**	44.5 (39.8)	**Clinical diagnosis**	**N° of patients (%)**
		Anosmia	44 (41.5%)
		Severe Hyposmia	11 (10.3%)
		Moderate Hyposmia	12 (11.3%)
		Mild Hyposmia	4 (3.8%)
		Normal	35 (33%)
**T1 olfactory score** **Mean (SD)**	57 (33.2)	**Clinical diagnosis**	**N° of patients (%)**
		Anosmia	16 (15.1%)
		Severe Hyposmia	29 (27.4%)
		Moderate Hyposmia	13 (12.3%)
		Mild Hyposmia	12 (11.3%)
		Normal	36 (34%)
**T2 olfactory score** **Mean (SD)**	72.4 (30)	**Clinical diagnosis**	**N° of patients (%)**
		Anosmia	9 (8.4%)
		Severe Hyposmia	12 (11.3%)
		Moderate Hyposmia	9 (8.4%)
		Mild Hyposmia	29 (27.4%)
		Normal	47 (44.3%)
**T0 gustatory score** **Mean (SD)**	1.9 (1.6)	**Clinical diagnosis**	**N° of patients (%)**
		Ageusia	30 (28.3%)
		Severe Hypogeusia	17 (16%)
		Moderate Hypogeusia	24 (22.6%)
		Mild Hypogeusia	5 (4.7%)
		Normal	30 (28.3%)
**T1 gustatory score** **Mean (SD)**	3 (1.1)	**Clinical diagnosis**	**N° of patients (%)**
		Ageusia	5 (4.7%)
		Severe Hypogeusia	3 (2.8%)
		Moderate Hypogeusia	21 (19.8%)
		Mild Hypogeusia	31 (29.2%)
		Normal	46 (43.4%)
**T2 gustatory score** **Mean (SD)**	3.4 (0.83)	**Clinical diagnosis**	**N° of patients (%)**
		Ageusia	2 (1.9%)
		Severe Hypogeusia	1 (0.9%)
		Moderate Hypogeusia	9 (8.5%)
		Mild Hypogeusia	38 (35.8%)
		Normal	56 (52.8%)

### Gustatory function evaluation results

At the first evaluation (T0), ageusia was detected in 30 cases (28.3%), 46 patients (43.3%) presented various degree hypogeusia while in 30 subjects (28.3%) the gustatory function was normal. The cases of ageusia (4.7%) decreased significantly at T1 control, while the number of patients who had recovered normal taste increased (43.4%). At the end of the observation period, the majority of patients had recovered a normal gustatory function (52.8%) while in 29.2% there was only a mild hypogeusia. Residual severe dysfunctions (i.e. ageusia or severe hypogeusia) were detected in 3 patients only. The results of the psychophysical gustatory evaluation are reported in Table 2.

### Statistical analysis results

**Fig. 1:**
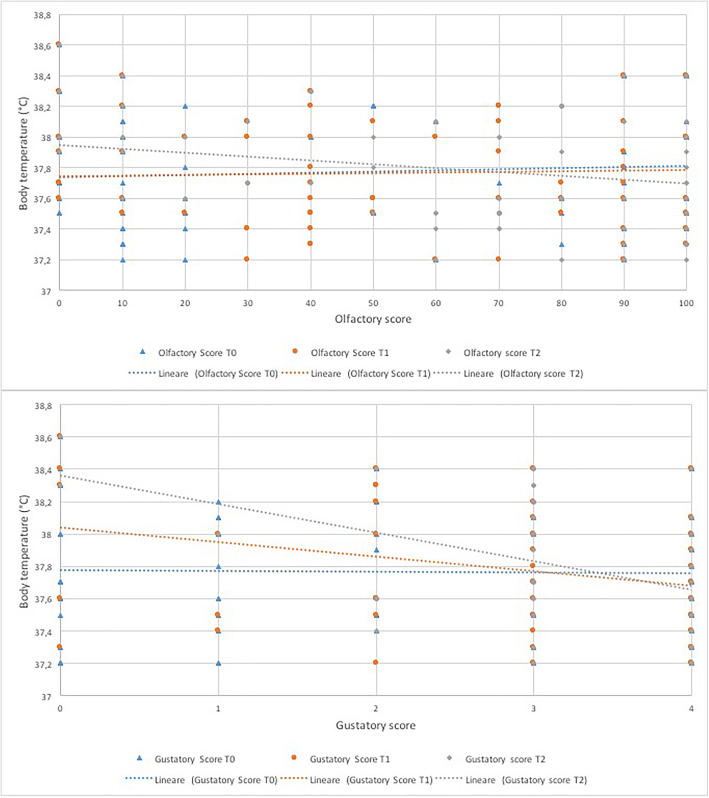
Linear regression curves representing the correlation between olfactory and gustatory scores and body temperature at each observation time

The linear regression analysis revealed a significant directly proportional correlation between the severity of the olfactory dysfunction and fever at T2 (B coefficient − 0.003, CI -0.005 – 0.000, *p* = 0.05) (Fig. 1) (Table 3). The same direct proportion was found between the severity of the gustatory dysfunction and the fever (Fig. 1) and the statistical correlation proved significant at T1 (B coefficient − 0.089, CI -0.158 – 0.021, *p* = 0.01) and T2 (B coefficient − 0.175, CI -0.261 – 0.090, *p* <  0.001) (Table 3).

**Table 3:** Linear regression analysis of the correlations between olfactory and gustatory scores at each observation time and the two assessed clinical variables

**Observation time**	**R**	**R**^**2**^	**B coefficient**	**95% confidence interval for B coefficient**		***P*value**
				**Lower limit**	**Upper limit**	
**DV: Body temperature IV: Olfactory score**						
**T0**	0.073	0.005	0.001	−0.001	0.003	**0.46**
**T1**	0.033	0.001	0.000	− 0.002	0.003	**0.73**
**T2**	0.191	0.036	- 0.003	−0.005	0.000	**0.05**
**DV: Body temperature IV: Gustatory score**						
**T0**	0.015	0.000	- 0.004	−0.053	0.045	**0.878**
**T1**	0.246	0.060	- 0.089	−0.158	−0.021	**0.01**
**T2**	0.369	0.136	−0.175	−0.261	− 0.090	**< 0.001**
**DV: Oxygen saturation IV: Olfactory score**						
**T0**	0.042	0.002	−0.002	−0.011	0.007	**0.67**
**T1**	0.082	0.007	−0.005	−0.026	0.006	**0.41**
**T2**	0.085	0.007	0.005	−0.007	0.018	**0.38**
**DV: Oxygen saturation IV: gustatory score**						
**T0**	0.135	0.018	0.162	−0.069	0.393	**0.17**
**T1**	0.107	0.011	0.185	−0.150	0.521	**0.28**
**T2**	0.368	0.136	0.835	0.425	1.245	**< 0.001**

*DV* dependent variable, *IV* independent variable

**Fig. 2:**
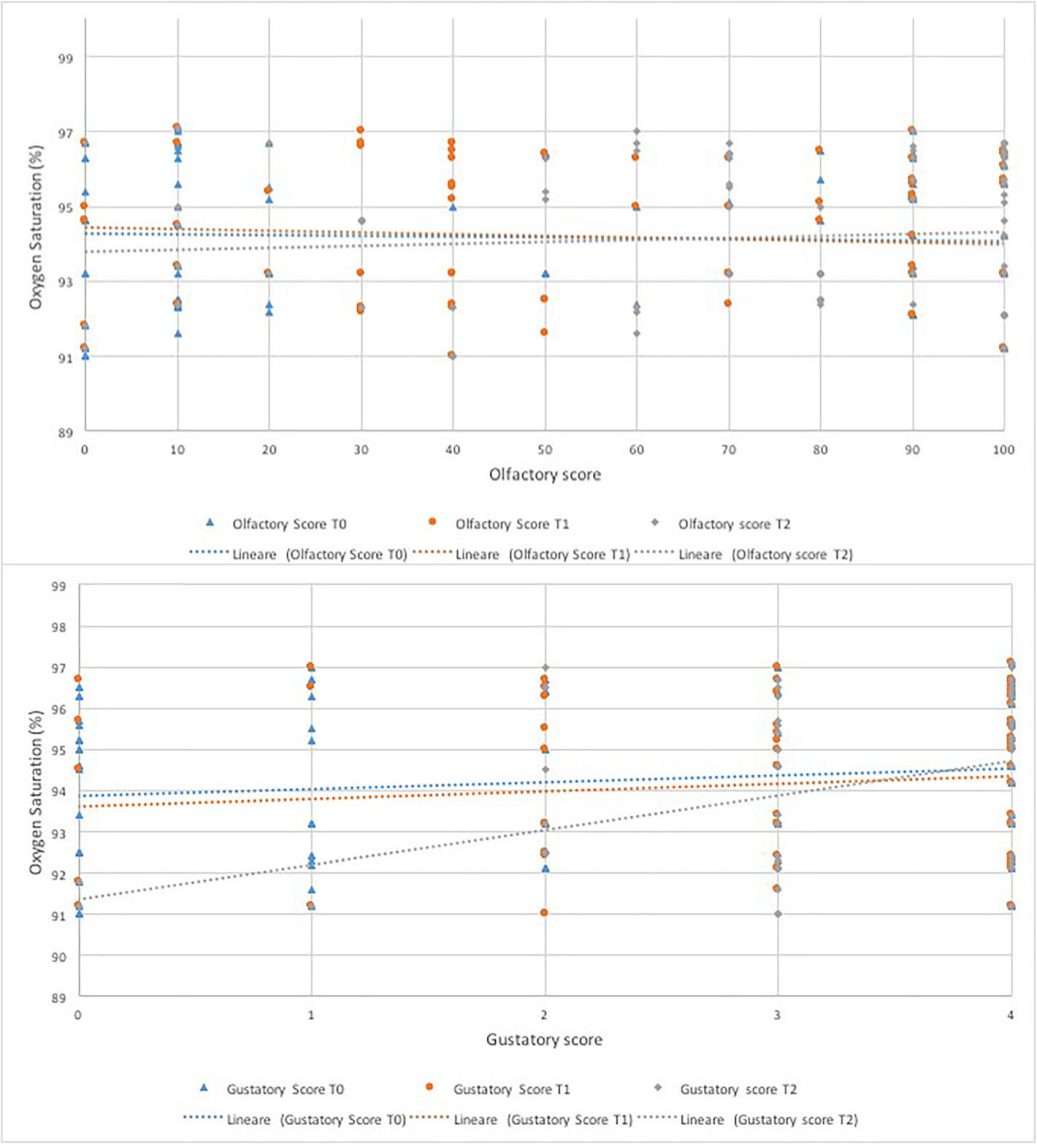
Linear regression curves representing the correlation between olfactory and gustatory scores and oxygen saturation at each observation time

The correlation between olfactory scores and the average oxygen saturation was not significant throughout the observation period (Fig. 2) (Table 3). A direct proportional relationship was found between the gustatory scores and the oxygen saturation with a significant correlation at T2 (B coefficient 0.835, CI 0.425–1.245, *p* <  0.001) (Fig. 2) (Table 3).

The logistic regression analysis detected significant correlation between the need for hospitalization and olfactory (Exp B 1.016, CI 1.002–1.030, *p* = 0.03) and gustatory (Exp B 2.227, CI 1.128–3.875, *p* = 0.005) scores at T2 (Table 4). However, by categorizing patients according to the severity of chemosensitive dysfunction, only the relationship between olfactory disturbance at T2 and hospitalization was significant (OR 3.750, CI 1.519–9.256, *p* = 0.005) (Table 5). On the contrary, the correlation between hospitalization and chemosensitive dysfunction at T0 was not statistically significant or clinically meaningful for either smell (OR 0.827, CI 0.347–1.970, *p* = 0.66) or taste (1.917, CI 0.730–5.036, *p* = 0.25). In contrast, the correlation between the presence of comorbidities and the in-patient course was highly significant (OR 4.286, CI 1.734–10.591, *p* = 0.002) (Table 5), in keeping with other studies, with patients with persistent chemosensory dysfunction more than four times more likely to require hospitalisation compared to those who had recovered.

**Table 4:** In-patient and out-patient group clinical characteristics and logistic regression analysis of the correlations between olfactory and gustatory scores at each observation time and the need for hospitalization

	**In-patient group**		**Out-patient group**		**Mann-Whitney U test** ***p*-value**		
**N° of patients** **N° (%)**	30 (28.3%)		76 (71.7%)				
**Body temperature** **Mean (SD)**	38.1 °C (0.36)		37.6 °C (0.34)		< 0.001		
**Oxygen saturation** **Mean (SD)**	92.5% (1.36)		94.9% (1.62)		< 0.001		
**Logistic regression analysis**							
**Observation time**	**Hospitalized group**	**Not-hospitalized group**	**B coefficient**	**Expected B coefficient**	**95% confidence interval for expected B coefficient**		***P*value**
					**Lower limit**	**Upper limit**	
**Olfactory score Mean (SD)**			**DV: Hospitalization IV: Olfactory score**				
**T0**	42.3 (38.2)	45.4 (38.1)	0.003	1.002	0.991	1.013	**0.72**
**T1**	52.7 (38.2)	58.7 (31)	0.006	1.006	0.993	1.018	**0.4**
**T2**	62 (36.6)	76.6 (26.1)	0.016	1.016	1.002	1.030	**0.03**
**Gustatory score Mean (SD)**			**DV: Hospitalization IV: Gustatory score**				
**T0**	1.6 (1.4)	2 (1.6)	0.186	1.205	0.912	1.591	**0.19**
**T1**	2.9 (1.1)	3 (1)	0.197	1.407	0.833	1.778	**0.31**
**T2**	2.9 (1.1)	3.5 (0.7)	0.801	2.227	1.280	3.875	**0.005**

*DV* dependent variable, *IV* independent variable

**Table 5:** Logistic regression and crosstabs analysis of the correlations between the presence of olfactory and gustatory dysfunctions at each observation time or comorbidities and the need for hospitalization

Observation time	**In-patient** **N° of patients (%)**	**Out-patients** **N° of patients (%)**	**Odds ratio**	**95% confidence interval for odds ratio**		***p*-value**
				**Lower limit**	**Upper limit**	
**Hospitalization / Olfactory dysfunction**						
**T0**						
Dysfunction group	18 (26.9%)	49 (73.1%)	0.827	0.347	1.970	**0.66**
Normal group	12 (30.8%)	27 (69.2%)				
**T1**						
Dysfunction group	18 (31%)	40 (69%)	1.350	0.572	3.184	**0.52**
Normal group	12 (25%)	36 (75%)				
**T2**						
Dysfunction group	15 (48.4%)	16 (51.6%)	3.750	1.519	9.256	**0.005**
Normal group	15 (20%)	60 (80%)				
**Hospitalization / Gustatory dysfunction**						
**T0**						
Dysfunction group	23 (32.4%)	48 (67.6%)	1.917	0.730	5.036	**0.25**
Normal group	7 (20%)	28 (80%)				
**T1**						
Dysfunction group	8 (27.6%)	21 (72.4%)	0.952	0.367	2.469	**> 0.99**
Normal group	22 (28.6%)	55 (71.4%)				
**T2**						
Dysfunction group	5 (41.7%)	7 (58.3%)	1.971	0.573	6.782	**0.31**
Normal group	25 (26.6%)	69 (73.4%)				
**Hospitalization / Comorbidities**						
Yes	16 (50%)	16 (50%)	4.286	1.734	10.591	**0.002**
No	14 (18.9%)	60 (81.1%)				

## Discussion

The attribution of a prognostic value to early and frequent COVID-19 symptoms such as the olfactory and gustatory dysfunctions, would be very useful in order to be able to design more efficient therapeutic pathways and better invest the resources of the national healthcare systems on those at highest risk of deterioration in this moment of crisis [21, 22]. Given the important implications that these studies may have on healthcare protocols and lay person behaviour, and in order to minimise recall bias, it is better to acquire data prospectively, which should then be analysed and offered to the public with great caution [16].

We were able to recruit 106 healthcare workers, who had rapid access to confirmatory testing, early in the course of their COVID-19 disease. In enrolling healthcare workers, we have excluded more elderly patients who are at higher risk of severe disease and those with significant comorbidities that prevent employment, however the mean age is older than that of the average age of the Italian population, and 28% of cases required hospitalisation, although there were no deaths. While there is a risk we have rejected any association between early olfactory and gustatory scores and clinical outcomes due to Type 2 error, we have sufficient power to detect a strong, statistically significant correlation between hospitalization and under-lying comorbidities, which are well-recognized risk factors for severity. We also found that persistence of anosmia at day 20 was associated with the need for hospitalization; we therefore believe our sample is adequate in size to address this question, however it would be important to further evaluate this in larger multicentre studies. This highlights the importance of prospective design in order to identify prognostic factors, and it is the first prospective study that evaluates the evolution of chemosensitive disorders during COVID-19 with psychophysical testing over time, and attempts to analyse the relationship with prognosis. Moreover, in prognostic studies, the observation period should start in the earliest stages of the disease to understand if it is possible to attribute an a priori prognostic value to a symptom. Previous studies have evaluated patients at different distances from the clinical onset [10, 12, 13, 15]. Generally, in the early stages of the disease the patient is in home quarantine; this makes their objective evaluation almost impossible. The use of the self-administered olfactory and gustatory psychophysical test [11] makes it possible to remotely evaluate these patients overcoming all the logistical problems. We chose 3 standardised time points so that we did not overly burden patients during the course of their illness, based on our previous studies showing that patients did not normally report improvement in smell and taste before 14 and 10 days respectively. We allowed the baseline score to occur within 4 days allowing for the time for swab results and to make contact with the patient, however we may have missed the peak of the severity of olfactory dysfunction.

Another fundamental characteristic for a prognostic, is the objectivity of both markers and the examined outcomes. We have highlighted the benefits of using psychophysical testing, and the validated home test kit made it feasible to study infected patients in the early course of disease when the transmission risk is highest [10, 12, 16].

While Sniffin Sticks and UPSIT tests are more widely used, they were unavailable at the outbreak of the pandemic in Italy, due to both supply and distribution issues. They are also expensive in comparison to the CCCRC test. By necessity, we therefore used the CCCRC as it can be produced in any hospital and therefore applied on a large scale. The CCCRC is a longer established smell test and indeed was used in validation studies of the newer tests, which have likely become more popular as they are ‘ready-made’. It most COVID studies of anosmia only the identification test of the Sniffin Sticks has been applied, and therefore the CCCRC test has the advantage of determining the olfactory threshold. The dilution of butanol allowed the creation of a home test kit, facilitating obtaining measurements during the critical stage of self-isolation. The home test was validated against in hospital testing produced slightly better threshold scores but slightly lower discriminatory scores such that when combined there was no significant difference in results based on the setting [11].

As yet, a universally agreed definition or scoring system to define severity of disease has not been agreed, although several have been proposed [17, 23]. In many studies the “need for hospitalization” has been used as proxy to distinguish between mild and moderate to severe disease, and so we also used logistic regression to assess for any association between severity of olfactory and gustatory dysfunction and hospitalization. However, thresholds for admission may vary between centres, or according to patient preference, as well as being determined by differing availability of healthcare resources across the duration of the pandemic. We therefore sought to include surrogate markers of severity that would not be prone to such bias, use the oxygen saturation as a marker of severity of respiratory disease and fever as a marker or severity of the immune response [17, 23]. We note that both responses might be influenced by other factors’, but we found that hospitalised patients had significantly higher temperatures and lower oxygen saturations than those patients managed at home.

The results of this study confirmed the high frequency of chemosensitive disorders in patients with SARS-CoV-2 infection and symptomatic COVID-19, as already reported by several other authors [3, 4, 7, 8, 10–15]. Although there is evidence of significant recovery, as reported in other studies, the functional recovery is more marked for taste than for smell (Table 2). In fact, a significant proportion of patients presented with severe residual olfactory disturbances even at the 20-day evaluation (Table 2). Longer studies will be required to assess for later recovery.

Overall, linear regression analysis found no statistical correlation between the severity of chemosensitive dysfunctions and the worsening of clinical outcomes (Figs. 1 and 2). In particular, in the earliest stages of the disease (T0) no significant correlation was found between the gustatory and olfactory scores and the final clinical outcome (Table 3). On the basis of this result, chemosensitive scores do not seem to have, in the early stages of the diseases, a prospective prognostic value which predicts the COVID-19 subsequent severity.

The correlation between olfactory and gustatory scores and fever was statistically significant in the advanced stages (T1 for smell, T1 and T2 for taste) of the observation period, while the relationship with oxygen saturation proved positive only with the taste score at T2 (Table 3), however the coefficient approached zero suggesting no meaningful relationship between variables. Consistent with what has been found in our previous retrospective studies [10, 12], it would seem that there may be a correlation between the persistence of chemosensitive disorders over time and the worsening of clinical outcomes. Long-lasting olfactory and gustatory disturbances may be related to the persistence of the virus in the upper respiratory tract and this could be the cause of a longer duration of fever in these patients [24].

Yan et al. [15] have previously reported association between self-reported anosmia and mild disease, defined as cases managed without hospitalization. They found that within a multivariable logistic regression model, self-reported olfactory loss was independently and inversely correlated with hospital admission. They further suggested that as olfactory dysfunction may be almost ubiquitous on psychophysical testing, the demonstration of olfactory dysfunction per se may not have prognostic value, but that the severity of loss may determine whether patients self-report and hold prognostic value with regard to hospitalization [25]. To explore this relationship in our series, a logistic regression analysis was performed between the olfactory and gustatory scores and the need for hospitalization which proved significant only at day 20, both for smell and taste (Table 4). We also analysed the patient cohort by dividing the patients into two groups: normal function (normal and mild dysfunctions) and severe dysfunction (moderate and severe functional reductions and anosmia or ageusia). The crosstab analysis and the Fisher’s exact test again found a direct statistical significant relation only between olfactory dysfunction severity at T2 and hospitalisation (OR 3.750, CI 1.519–9.256, *p* = 0.005); that is that persistence of olfactory loss associates with more severe disease but chemosensitive scores at the onset of COVID-19 do not.

The pathophysiological mechanism underlying chemosensitive dysfunctions in COVID-19 has yet to be fully elucidated [24, 26]. What determines the severity of clinical presentation of COVID-19 remains equally mysterious. As expression of both ACE2 and TMPRSS2 receptors have been shown to increase with age in a mouse model, this has been proposed to account for the increased susceptibility seen in older age groups [27]. Further speculation surrounds whether levels of expression of the entry receptors may influence severity of both the disease itself and chemosensitive disturbances. It is hard to imagine a mechanism where the severity of anosmia and the disease in general would be inversely associated, as one might expect a greater degree of damage to the olfactory epithelium or greater neuroinvasion in the setting of upregulated entry receptor expression. Indeed, this would be consistent with our finding of longer duration of olfactory dysfunction associating with more severe disease.

## Conclusions

In conclusion, based on the results of this study, the severity of olfactory and gustatory dysfunctions at the onset of disease do not associate with the severity of the COVID-19 course, although larger, prospective studies are required for confirmation. Multicentre, large collaborative effort is now required to address remaining questions regarding COVID-19 and its impact on sense of smell and taste. At the present time, our findings suggest that loss of smell and taste should not be overlooked in the belief that they are prognostic signs of mild disease.

## Data Availability

Full data may be made available for analysis by contacting the first author.
